# *In Vitro* Binding Capacity of Bile Acids by Defatted Corn Protein Hydrolysate

**DOI:** 10.3390/ijms12021066

**Published:** 2011-02-08

**Authors:** Jauricque Ursulla Kongo-Dia-Moukala, Hui Zhang, Pierre Claver Irakoze

**Affiliations:** State Key Laboratory of Food Science and Technology, Jiangnan University, Lihu Road 1800, Wuxi 214122, Jiangsu, China; E-Mails: kjauricque@yahoo.fr (J.U.K.-D.-M.); irakozefr@yahoo.fr (I.P.C.)

**Keywords:** defatted corn protein, enzymatic hydrolysis, Flavourzyme hydrolysate, bile acid binding capacity

## Abstract

Defatted corn protein was digested using five different proteases, Alcalase, Trypsin, Neutrase, Protamex and Flavourzyme, in order to produce bile acid binding peptides. Bile acid binding capacity was analyzed *in vitro* using peptides from different proteases of defatted corn hydrolysate. Some crystalline bile acids like sodium glycocholate, sodium cholate and sodium deoxycholate were individually tested using HPLC to see which enzymes can release more peptides with high bile acid binding capacity. Peptides from Flavourzyme defatted corn hydrolysate exhibited significantly (*p* < 0.05) stronger bile acid binding capacity than all others hydrolysates tested and all crystalline bile acids tested were highly bound by cholestyramine, a positive control well known as a cholesterol-reducing agent. The bile acid binding capacity of Flavourzyme hydrolysate was almost preserved after gastrointestinal proteases digestion. The molecular weight of Flavourzyme hydrolysate was determined and most of the peptides were found between 500–180 Da. The results showed that Flavourzyme hydrolysate may be used as a potential cholesterol-reducing agent.

## 1. Introduction

Protein is a food substance essentially required by the body that cannot be substituted by other compounds in the whole body tissues for its nitrogen contents; hence it must be provided in food. The population in developing countries generally obtains protein from cereals and one of the cereals types used as a rice substitute is corn [1]. Each corn variety contains a different amount of protein of differing quality. Protein quality is determined by the protein concentration and its amino acid patterns, and each cereal has a different amino acid composition [2].

Defatted corn, a byproduct of the corn oil industry, is rich in proteins, minerals and B-group vitamins, and has a balanced amino acid composition, particularly the content of lysine; sulfur-containing amino acids and tryptophan are equivalent to those proposed by WHO/FAO for reference protein [3–5].

Protein is required for normal growth, production, and health, thus an ideal protein must possess an amino acid composition in line with the human and animal requirements. Peptides derived from *in vivo* digestion of whole proteins by hydrolyzation with bacterial proteases before ingestion have been reported to carry specific bioactivities. The identification and synthesis of these bioactive peptides has received considerable attention in recent years [6–8].

Numerous studies have demonstrated that peptides may influence bile acids and cholesterol metabolism. Bile acids are the major metabolites of cholesterol and facilitate its elimination in the feces by the formation of micelles that solubilize the cholesterol in the bile. Within the intestinal lumen, bile acids interact with lipases and assist the lipolysis and absorption of fats, including fat soluble vitamins. In healthy people, only small quantities of bile acids are found in the peripheral circulation and urine [9]. Thus, depletion of bile acids from the circulation would promote cholesterol conversion into additional bile acids, thereby leading to significant reductions of liver and serum LDL cholesterol levels [10].

Many investigations have focused on peptides with angiotensin converting enzyme (ACE) inhibitory and antioxidant effects [11]. Bile acid binding by dietary fiber is well reported. Thus, various studies have been carried out on bile acid binding from different materials such as corn bran dietary fiber [12], extruded potato peels [13], chitosan [14], soy bean, black eye bean, garbanzo and lima bean; kidney bean, black gram, bengal gram and moth bean [15,16].

However, few reports are available on peptides with bile acid binding capacity. Some researchers reported the involvement of post-digestion hydrophobic peptides in plasma cholesterol-lowering effect of dietary plant proteins [17], bile acid binding activity of buckwheat protein [18] and the binding of bile acids by lupin protein isolates and their hydrolysates [19].

To date, not a single research on bile acid binding peptides from defatted corn protein has been reported. Some studies have found that protein hydrolysate has higher binding potential than the protein itself [20,21].

The present work was undertaken to determine the best enzyme for hydrolyzing defatted corn protein with the purpose of generating more peptides with higher bile acid binding capacity. In addition, the stability after gastrointestinal protease digestion as well as amino acid content and molecular weight of hydrolysate were investigated. The defatted corn protein hydrolysis by proteases could suggest its potential application as a cholesterol-reducing agent for hypercholesterolemic patients.

## 2. Results and Discussion

### 2.1. Enzymatic Hydrolysis of Defatted Corn Protein by Five Proteases

In the present study, defatted corn proteins were independently hydrolyzed with Alcalase, Neutrase, Trypsin, Protamex and Flavourzyme in order to determine the most suitable enzyme for the production of bile acid binding peptide.

**Figure 1.**
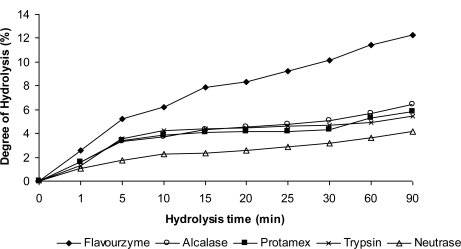
Hydrolysis curves of defatted corn protein with five different protease preparations.

The hydrolysis curves of defatted corn protein with the five enzymes after 90 min of reaction are shown in Figure 1. All the curves showed a high rate of enzymatic hydrolysis during the first 15 min and then the rate of hydrolysis gradually declined with time. This was similar to previous findings [22,23]. Adler-Nissen [24] attributed the reduction in the rate of hydrolysis to the competition between unhydrolyzed protein and the peptides being constantly formed during hydrolysis.

The results showed that the degree of hydrolysis (DH) of defatted corn protein with Flavourzyme was 12.25% and was significantly higher than those of Alcalase, Protamex, Trypsin and Neutrase with 6.40, 5.84, 5.45, and 4.20% respectively (Table 5). The high DH expressed by Flavourzyme produced by *Aspergillus orizae* may be related to the complex properties as it possess some endoproteinase and exopeptidase abilities [25].

### 2.2. *In Vitro* Binding Capacity

Three bile acids were used to test the *in vitro* binding capacity of different hydrolysates comparatively to that of cholestyramine.

#### 2.2.1. Binding Capacity of Sodium Glycocholate

**Figure 2.**
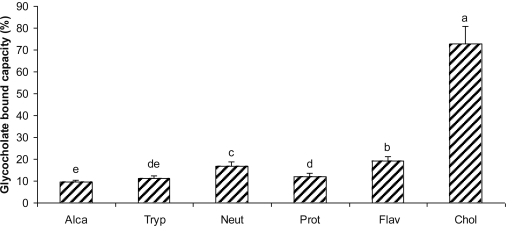
Sodium glycocholate binding by peptides from five differents hydrolysates of defatted corn protein. Alca: Alcalase; Tryp: Trypsin; Neut: Neutrase; Prot: Protamex; Flav: Flavourzyme. Cholestyramine (Chol) was used as a positive control. Different letters indicate significant differences (*p* < 0.05).

As shown in Figure 2, all the hydrolysates resulting from the five enzymes were capable of binding sodium glycocholate. Sodium glycocholate was bound by hydrolysate from Alcalase, Trypsin, Neutrase, Protamex, and Flavourzyme to the degree of 9.53, 11.16, 16.80, 12.15 and 19.01%, respectively. Cholestyramine was used as a positive control and displayed 72.77% glycocholate binding capacity. Hydrolysate from Alcalase showed a significantly lower glycocholate binding capacity while the value for the hydrolysate from Flavourzyme was significantly higher than those of the other hydrolysates. Cholestyramine bound sodium glycocholate and taurocholate by 87% and 93%, respectively [26]. Story and Krichevsky [27] reported that cholestyramine bound glycocholate under various conditions to a degree of 74.2%. In our study, cholestyramine binding by glycocholate was similar to that observed by Story and Krichevsky [27]. Alfalfa and wheat bran have been shown to have a glycocholate binding capacity of 11.5% [27] and 100% [28], respectively; and the defatted soybean hydrolysate 0.2 to 8.5% [19]. Hydrolysate from Flavourzyme showed higher glycocholate binding capacity than alfalfa and defatted soybean hydrolysate.

#### 2.2.2. Binding Capacity of Sodium Cholate

**Figure 3.**
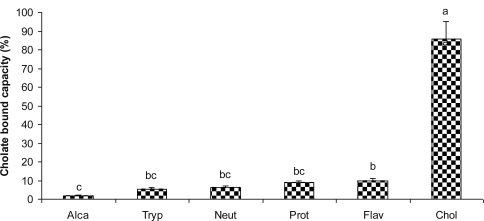
Sodium cholate binding by peptides from five differents hydrolysates of defatted corn protein. Alca: Alcalase; Tryp: Trypsin; Neut: Neutrase; Prot: Protamex; Flav: Flavourzyme. Cholestyramine (Chol) was used as a positive control. Different letters indicate significant differences (*p* < 0.05).

Sodium cholate was bound by hydrolysate from Alcalase, Trypsin, Neutrase, Protamex, and Flavourzyme to the degree of 1.93, 5.52, 6.26, 8.82 and 9.99%, respectively, while cholestyramine, used as a positive control, had 85.78% cholate binding capacity (Figure 3). These values are much lower than those recorded with sodium glycocholate.

Hydrolysate from Alcalase showed significantly (*p* < 0.05) lower cholate binding capacity than cholestyramine, which has been reported to bind sodium cholate to the degree of 80% [13] and 60.7% [27]. Camire and Camire *et al.* [13,28] reported cholate binding by cholestyramine, three types of raisins, wheat bran and various types of potato peels at a cholate concentration of 12.5 mM. Their work indicated cholate binding of 75%, 15–20%, 10% and 1.9–8.1%, respectively. Hydrolysate from Flavourzyme exhibited higher cholate binding capacity than potato peels.

#### 2.2.3. Binding Capacity of Sodium Deoxycholate

**Figure 4.**
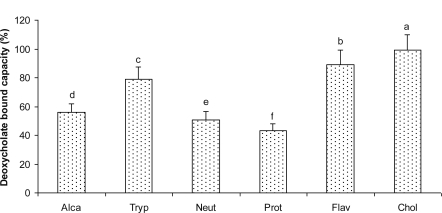
Sodium deoxycholate binding by peptides from five differents hydrolysates of defatted corn protein. Alca: Alcalase; Tryp: Trypsin; Neut: Neutrase; Prot: Protamex; Flav: Flavourzyme. Cholestyramine (Chol) was used as a positive control. Different letters indicate significant differences (*p* < 0.05).

Under similar condition, all hydrolysates from defatted corn protein hydrolyzed by proteases showed higher percentage of bile acid binding with sodium deoxycholate than sodium glycocholate and sodium cholate (Figure 4). Sodium deoxycholate was bound by hydrolysate from alcalase to a degree of 55.76%, 78.68% by hydrolysate from Trypsin, 50.77% by hydrolysate from Neutrase, 43.40% by hydrolysate from Protamex, 86.90% by hydrolysate from Flavourzyme and 99.15% by cholestyramine. The hydrolysate from Flavourzyme had the highest binding capacity as compared to the other hydrolysates. Cholestyramine has been shown by other researchers to bind sodium deoxycholate by 99% [13], 92.5% [27] and 85% [28]. Our result was similar to that observed by Camire *et al.* [13]. Protein isolates F and its hydrolysate [19], Alfalfa [27], raisins, wheat bran [28], and potato peels [13] showed 58.4–69.5%, 10.8%, 5–10%, 15%, and 10.6–18.9%, respectively. Compared to these samples, our hydrolysates from Flavourzyme showed a much higher deoxycholate binding capacity.

As demonstrated by previous works [14,29–32], our investigations confirmed that bile acid binding degree decreases with hydroxyl group increment in steroid ring structure. In addition, we found that sodium cholate primary bile acid (three hydroxyl) was the less bound with hydrolysate from Alcalase than sodium glycocholate, a conjugation of sodium cholate with glycine; however, sodium deoxycholate secondary bile acid (two hydroxyl) was the best bound. Numerous authors [18,33,27] have reported a greater binding capacity of sodium deoxycholate than sodium cholate. Kern *et al.* [34] proposed that less sodium cholate, a trihydroxy bile acid, was bound than dihydroxy bile acid because hydrophobic interactions are involved with binding.

The method reported here appears to be a satisfactory technique for the measurement of the bile acid binding capacity of peptides. Cholestyramine, as expected, bound all the bile acids selected under the same treatment. The concentration of bile acids used and the percentage bound in the present study are in accordance with findings from previous studies. However, there is no obvious correlation between bile acid binding and the degree of hydrolysis. As the results showed, hydrolysate obtained with Flavourzyme contained effective peptides capable to bind bile acids. This could be explained by the presence of Flavourzyme acting as an endoprotease and exopeptidase, providing a broader specificity to release peptides rich in hydrophobic amino acid residues. These hydrophobic amino acids can bind bile acids strongly via hydrophobic reactions, since the hydrophobic amino acids present a strong interaction with lipids (cholesterol, bile acids, others sterols and others lipids). Moreover, higher bile acid binding by Flavourzyme hydrolysate in our studies may be due to the use of physiological pH of 6.5, which is closer to the pHi of the hydrophobic amino acids. The results given in the literature have shown that the binding of bile acid mixture, simulating the condition in the human body, occurred in a noncompetitive manner. Despite the need for bile acid mixture analysis, Flavourzyme hydrolysate could be considered as a suitable natural compound for bile acid binding.

Bile acids, especially deoxycholate, are thought to be involved in the etiology and development of colorectal cancer [35]. The synthesis of bile acids from cholesterol is adjusted by their concentration in the liver [36]. The presence of Flavourzyme hydrolysate in the intestine can decrease not only enterohepatic circulation of bile acids, but also their contact with colorectal mucosa through the binding effect. Therefore, it is possible for humans to ingest some Flavourzyme hydrolysate to prevent hypercholesterolemia and colorectal cancer at the same time.

### 2.3. Stability of Bile Acid Binding by Flavourzyme Hydrolysate after Digestion by Gastrointestinal Proteases

The stability of Flavourzyme hydrolysate against gastrointestinal proteases *in vitro* was tested in order to predict the bile acid binding *in vivo*. Some food protein derived-bioactive-peptides, for example ACE-inhibitory peptides, failed to express bioactivity after oral administration to spontaneously hypertensive rats due to the fact that they were hydrolyzed in the gastrointestinal tract to peptides or amino acids with reduced activity [37]. The bile acid binding of Flavourzyme hydrolysate was almost preserved after gastrointestinal proteases treatment (Table 1), suggesting that these hydrolysates may be resistant to digestion in the gastrointestinal tract. Previous reports have also shown that small peptides have low susceptibility to hydrolysis by gastric proteases [38,39].

### 2.4. Total Amino Acid Analysis

Flavourzyme hydrolysate contains all the essential amino acids in good proportion (Table 2). The predominant amino acids amongst the essential amino acids were Leucine, Valine and Phenylalanine, while those amongst the hydrophobic amino acids were Leucine, Valine, Alanine, Proline, Glycine and Phenylalanine. Hydrophobicity of the peptides may increase interaction of such peptides with fatty acids and hence confer the peptide’s bile acid binding capacity. It was suggested that most naturally occurring hypocholesterolemic peptides contain Tryptophan, Glycine, Isoleucine, Alanine, Lysine and Proline [40,41], and that they are mostly hydrophobic. Therefore, the bile acid binding capacity of the Flavourzyme hydrolysate seemed to be caused by these amino acids in the peptides. Moreover, the bile acid binding capacity of the Flavourzyme hydrolysate depended upon the amino acid sequence of the peptides. Furthermore, Flavourzyme hydrolysate has a well balanced amino acid composition and its ratio of amino acids is close to that of protein quality for adults recommended by the Food and Agricultural Organization/World Health Organization reference [42]. Therefore, Flavourzyme hydrolysate not only showed good bile acid binding capacity but also good nutritive value.

### 2.5. Free Amino Acids Content

The total amount of free amino acids in the Flavourzyme hydrolysate was 5.17 g/100 g, with a predominance of hydrophobic amino acids (Table 3). Morita *et al.* [43] reported that Methionine in the diet plays a central role in cholesterol-lowering effects through the regulation of Apo A–I secretion from the liver into the blood circulation. The release of free amino acids is theoretically inevitable at elevated DH, and it is also a property of the enzyme and its hydrolysis properties on a substrate [44]. This is in conformity with our finding and confirmed that the low free amino acids content of the Flavourzyme hydrolysate was due to Flavourzyme protease properties. High free amino acids content in hydrolysates can affect their functionality, e.g., hydrolysates produced as nutritional supplements with high free amino acids composition have been demonstrated to have low biological value [45]. They are absorbed at a lower rate in the digestive tract, as the osmotic pressure of free amino acids is higher than that of peptides.

### 2.6. Molecular Weight Distribution

The molecular weight distribution of Flavourzyme hydrolysate ranged from 133 Da to more than 10000 Da, with most of the peptides being between 500–180 Da (Table 4). The unhydrolysed protein showed molecular weight fractions of 15,236 Da. The presence of low molecular weight fractions indicate that the hydrolysate could comprise of peptides having five or less amino acids residues. In addition, the function of protein hydrolysate was related to the molecular weight distribution as well as the amino acid present [46,47]. The cleavage of peptide bonds leads to an increase in the concentration of free amino and carboxyl groups, which increases solubility. Hydrolysis also disrupts the protein tertiary structure and reduces the molecular weight of the protein and, consequently, alters the functional properties of proteins [24,48]. However, extensive hydrolysis could have a negative impact on the functional properties [48].

## 3. Materials and Methods

### 3.1. Materials

Defatted corn meal was obtained from China Corn Oil Company Ltd., Shandong Province, P.R. China. Cholestyramine and sodium cholate were purchased from International Laboratory USA (Nanjing, P.R. China), sodium glycocholate, horse heart cytochrome C (12,400 Da), bacitracin (1450 Da), gly-gly-tyr-Arg (451 kDa) were purchased from Sigma Chemical Co. (St. Louis, USA) and gly-gly-gly (189 Da) was purchased from Fluka (Japan). Enzymes used were: Alcalase 2.4 L, Trypsin, Neutrase 2.4 L, Protamex and Flavourzyme, all provided by Novo Nordisk (Bagsvaerd, Denmark). All the chemicals used were of analytical grade and purchased from Sinopharm Chemicals Reagent Company (SCRC), Shanghai, China.

### 3.2. Methods

#### 3.2.1. Preparation and Hydrolysis of Defatted Corn Protein

Defatted corn proteins isolates were prepared by alkaline extraction at pH 11.50, followed by precipitation at its isoelectric point of pH 4.50, and freeze-dried. The freeze-dried defatted corn protein was dissolved in distilled water at a concentration of 5% (w/v) before hydrolysis with five different enzymes independently. Homogenization was carried out for each enzyme for 30 min in order to adjust the pH (through addition of 0.5 M NaOH) and temperature to the appropriate values. After the optimum condition was reached, the reaction was initiated by adding each of the five different enzymes with continuous stirring. Hydrolysis was carried out for 90 min and the pH of the mixture was kept constant by adding 0.5 M NaOH solution continuously to the reaction mixture. The amount of alkali added to keep the pH constant was recorded and used to calculate the degree of hydrolysis (DH). Then, the mixture was heated at 85 °C for 10 min to inactivate the enzyme and centrifuged by freezing centrifugation (ZOPR-52D, Hitachi Koki Co., Japan) at 10,000 rpm for 25 min. The supernatant was freeze-dried and stored in a desiccator for further use.

#### 3.2.2. Degree of Hydrolysis (*DH*): pH-Stat Method

Degree of hydrolysis (*DH*) was defined as the percentage ratio between the number of peptide bonds cleaved *(h*) and the total number of peptide bonds in the substrate studied (*h*_tot_). *DH* was evaluated by pH-stat method which allowed the estimation of *DH* based on the consumption of alkali to maintain a constant pH at the desired value. The degree of hydrolysis was calculated using the following equation (1) [24]:

(1) $$\text{DH}(\%)=V_{\text{b}}\times N_{\text{b}}\times \frac{1}{\alpha }\times \frac{1}{M_{\text{p}}}\times \frac{1}{h_{\text{tot}}}\times 100$$

where *V*_b_ is the amount of alkali consumed in mL; *N*_b_ is the normality of the alkali; *M*_p_ is the mass of protein (*N* × 6.25) in g; *h*_tot_ is the total number of peptide bonds in the protein substrate (9.2 meqv/g defatted corn protein) and, α is the average degree of dissociation of the α-NH_2_ groups released during hydrolysis. The *DH* of the five defatted corn protein hydrolysates can be seen in Table 5.

#### 3.2.3. Bile Acid Binding Assay

The *in vitro* bile acid binding was executed through modification of the procedure previously by Hu *et al.* [12]. Each bile acid (as substrate) was dissolved in 50 mmol/L phosphate buffer (pH 6.5) to make a 2 mM bile acid solution, which is in the same range of bile acid concentration in the human body (1.5–7 mM), and the pH was simulated to the physiological pH of the duodenum. Ten milligrams of the hydrolysate sample was added to each tube containing one milliliter of bile acid solution, and the individual substrate solution without samples was used as blank. Then tubes were incubated for one hour at 37 °C in a shaking water bath. Mixtures were centrifuged at 10 °C with 10,000 rpm for 30 min in an ultracentrifuge (Model J-26XPI, Beckman, USA). The supernatant was removed into a second set of tubes and frozen at −20 °C for bile acid analysis. Bile acids were analyzed using HPLC (Model 1525, Waters, USA) on a symmetry C18 column (3.9 × 150 mm i.d., 5 μm particle size, Waters, USA), maintained at 30 °C. The injected sample volume was 10 μL for each bile acid analysis. The bile acid was eluted with 40% acetonile + 60% of 0.05% H_3_PO_4_ at a flow rate of 1 mL/min for 24 min. The absorbance of the eluate was monitored continuously at 210 nm (Model 2996 PDA detector, Waters, USA) and quantitated using standard calibration curves generated from the peak area responses of the standard solutions. Duplicate assays were conducted for each bile acid essay.

The percentage of bound bile acid was calculated as bound (%) = [(*C*_c_ − *C*_s_)/*C*_c_] × 100, where *C*_c_ and *C*_s_ represent bile acid concentrations in the control and in samples, respectively.

DH and bile acid binding capacity obtained from Flavourzyme hydrolysate were significantly higher in comparison to hydrolysate obtained from Alcalase, Trypsin, Neutrase and Protamex. Therefore, further experiments were focused on Flavourzyme hydrolysate.

#### 3.2.4. Stability against Gastric Protease *in Vitro*

The stability against *in vitro* gastric proteases was assessed based on the method described by Wu and Ding [49]. A 1% (w/v) hydrolysate solution in 0.1 M KCl–HCl buffer (pH 2.0) was treated with pepsin for 4 h in a rotary water bath at 37 °C. The pepsin-treated peptide was heated to boiling in a water bath for 15 min then adjusted to pH 7.0 with addition of 2 N NaOH. A 1 mL aliquot of the neutralized suspension was centrifuged at 10,000 g for 40 min and the supernatant portion was used to determine bile acid binding. The remaining portion of the suspension was further digested by 2% (w/w) pancreatin at 37 °C for 4 h, followed by enzyme inactivation by boiling for 15 min. The reaction solution was centrifuged at 10,000 g for 40 min. The supernatant portion was used for additional testing on bile acid binding capacity.

#### 3.2.5. Total Amino Acids Analysis

The dried Flavourzyme hydrolysate (100 mg) was subjected to acid hydrolysis with 5 mL of 6 M HCl under nitrogen atmosphere for 24 h at 110 °C. The hydrolysate was washed into a 50 mL volumetric flask and made up to the mark with distilled water. The amino acids were subjected to RP-HPLC analysis (Agilent 1100, USA) after precolumn derivatization with *o*-phthaldialdehyde (OPA). Amino acid composition was expressed as g amino acid per 100 g protein.

#### 3.2.6. Free Amino Acids Content

The dried Flavourzyme hydrolysate (1 g) was dissolved in 5% (w/v) TCA (25 mL) and centrifuged at 10,000 g for 10 min. The clear supernatant containing mainly free amino acids and some short peptides were derivatized by o-phthaldialdehyde (OPA) followed by reversed phase high performance liquid chromatography (RP-HPLC) analysis, carried out in an Agilent 1100 (Agilent Technologies, Palo Alto, CA, USA). Sample (1 mL) was injected on a Zorbax 80A C18 column (4.6 i.d. × 180 mm, Agilent Technologies, Palo Alto, CA, USA) at 40 °C with detection at 338 and 262 nm. Mobile phase A was 7.35 mmol/L sodium acetate/triethylamine/tetrahydrofuran (500:0.12:2.5, v/v/v), adjusted to pH 7.2 with acetic acid, while mobile phase B (pH 7.2) was 7.35 mmol/L sodium acetate/methanol/acetonitrile (1:2:2, v/v/v). The amino acid composition was expressed as g of amino acid per 100 g of protein. The results were processed with the aid of ChemStation for LC 3D software (Agilent Technologies, Palo Alto, CA, USA).

#### 3.2.7. Molecular Weight Distribution

Molecular weight distribution was determined using a Waters^TM^ 600E Advanced Protein Purification System (Waters Corporation, Milford, MA, USA). A TSK gel 2000SWXL (7.8 × 300 mm) column with 10% acetonitrile + 0.1% TFA in HPLC grade water as the mobile phase.

The calibration curve was obtained by running horse heart cytochrome C (12,400 Da), bacitracin (1450 Da), gly-gly-tyr-Arg (451 kDa) and gly-gly-gly (189 Da). The results obtained were processed with the aid of Millennium^32^ Version 3.05 software (Waters Corporation, Milford, MA 01757, USA).

#### 3.2.8. Statistical Analysis

All analyses were carried out in duplicate and data were presented as mean ± SD. Analysis of variance (ANOVA) was carried out using SAS package (SAS Institute, Cary, NC). Comparisons between means were done using a Duncan’s multiple-range test with a probability of *p* < 0.05.

## 4. Conclusion

This study demonstrated that bile acid binding peptides can be generated from defatted corn protein by enzymatic hydrolysis. Defatted corn protein is a good source of bile acid binding peptides when hydrolyzed with Flavourzyme, resulting in a large proportion of low-molecular-weight (500–180 Da) peptides. As expected, defatted corn protein hydrolysed with Flavourzyme shifted the molecular weight distribution to lower values. Moreover, the bile acid binding capacity of Flavourzyme hydrolysate was almost preserved after *in vitro* incubation with gastric proteases, suggesting that the Flavourzyme hydrolysate may be resistant to digestion in the gastrointestinal tract. The defatted corn hydrolysate prepared with Flavourzyme might be utilized to develop physiologically functional food with bile acids binding capacity.

The defatted corn protein hydrolysates thus have excellent applications for future product development by virtue of their functional properties.

## Figures and Tables

**Table 1.** Bile acid binding capacity after digestion by gastrointestinal proteases.

**Protease**	**Glycocholate**	**Cholate**	**Deoxycholate**
Control	19.01 ± 0.36 ^a^	9.99 ± 1.65 ^a^	86.90 ± 0.28 ^a^
Pepsin	17.86 ± 0.70 ^a^	8.10 ± 0.42 ^a^	79.63 ± 1.24 ^b^
Pepsin + pancreatin	18.84 ± 0.26 ^a^	8.74 ± 0.60 ^a^	83.04 ± 0.40 ^a^

Each measurement was carried out in duplicate. Values with the same letters denote no significant difference (*p* < 0.05).

**Table 2.** Amino acid composition (g/100 g) of Flavourzyme hydrolysate.

**Amino acid**	**Amount**
Aspartic acid	5.13
Glutamic acid	8.22
Serine	4.26
Histidine	2.8
Glycin	4.13
Threonine	3.63
Arginine	1.44
Alanine	5.15
Tyrosine	3.48
Cysteine	0.24
Valine	5.40
Methionine	0.64
Phenylalanine	4.12
Isoleucine	3.63
Leucine	6.12
Lysine	1.13
Proline	5.10
Tryptophan	1.72
**Total**	**66.34**

**Table 3.** Free amino acids content (g/100 g) of Flavourzyme hydrolysate.

**Free amino acids**	**Amount**
Aspartic acid	0.04
Glutamic acid	0.14
Serine	0.04
Histine	0.10
Glycin	0.14
Threonine	0.20
Arginine	0.06
Alanine	0.26
Tyrosine	0.40
Cysteine	0.08
Valine	0.52
Methionine	0.16
Phenylalanine	0.80
Isoleucine	0.40
Leucine	1.64
Lysine	0.16
Proline	0.028
Total	5.17
Hydrophobic amino acids	4.71

**Table 4.** The molecular weight distribution of Flavourzyme hydrolysate from defatted corn protein.

**Molecular Weight (Da)**	**Amount (%Area)**
>10000	19.04
10000–5000	12.51
5000–3000	7.96
3000–2000	6.05
2000–1000	11.46
1000–500	13.69
500–180	23.24
<180	6.06

**Table 5.** Optimum conditions of proteases and the degree of hydrolysis (*DH*) of five defatted corn protein hydrolysates.

**Enzyme**	**Conditions**			
	**Temperature (°C)**	**pH Value**	**Hydrolysis Time (min)**	**Degree of Hydrolysis (%*DH*)**
Alcalase	60	8.5	90	6.40 ± 0.14 ^b^
Trypsin	50	7.5	90	5.45 ± 0.35 ^bc^
Neutrase	50	7.0	90	4.20 ± 0.43 ^c^
Protamex	55	7.0	90	5.84 ± 0.79 ^bc^
Flavourzyme	50	6.0	90	12.25 ± 1.20 ^a^

Each measurement was carried out in duplicate. Values with the same letters denote no significant difference (*p* < 0.05).
